# Supplementary material for: Plasmonic nanocavity-enabled universal detection of layer-breathing vibrations in two-dimensional materials
Source: Light Sci Appl. 2026 Feb 6;15:109. doi: 10.1038/s41377-026-02203-x (PMC12880968; doi:10.1038/s41377-026-02203-x)
Supplement: Supplementary file 1 — SUPPLEMENTAL MATERIAL for Plasmonic nanocavity-enabled universal detection of layer-breathing vibrations in two-dimensional materials [file 41377_2026_2203_MOESM1_ESM.pdf]

Supplementary information for:
Plasmonic nanocavity-enabled universal detection of layer-breathing vibrations in two-dimensional materials

Heng Wu,[1,2,*] Miao-Ling Lin,[1,2,*] Sen Yan,[3] Lin-Shang Chen,[1] Zhong-Jie Wang,[1,2] Yi-Fei Zhang,[1,2] Ti-Ying Zhu,[1,2] Zheng-Yu Su,[1] Jun Wang,[3] Xue-Lu Liu,[1] Zhong-Ming Wei,[1,2] Yan-Meng Shi,[1,2] Xiang Wang,[3] Bin Ren,[3] and Ping-Heng Tan[1,2,†]

[1]*State Key Laboratory of Semiconductor Physics and Chip Technologies, Institute of Semiconductors, Chinese Academy of Sciences, Beijing 100083, China*

[2]*Center of Materials Science and Optoelectronics Engineering, University of Chinese Academy of Sciences, Beijing 100049, China*

[3]*State Key Laboratory of Physical Chemistry of Solid Surfaces, College of Chemistry and Chemical Engineering, Xiamen University, Xiamen 361005, China*

[*] These authors contributed equally to this work.

[†] phtan@semi.ac.cn

CONTENTS

I. Supplementary Figures 3

II. Supplementary Notes 7

1. Background signal subtraction 7
2. Linear chain model for 2DMs coupled with AuNCs 8
3. Derivation of Raman tensor elements for 2DMs coupled with AuNCs 8
4. Possible enhancement effect of LB modes in AuNCs/NLG by out-of-plane components of local fields 10
5. Plasmon-enhanced LB modes in NLG coupled with AuNCs with different shapes and densities 11
6. Determine the layer number of hBN and LB force constants in AuNCs/NL-hBN structure 15
7. Simulation of LB modes in t$(m+m)$LG based on IBPM 16

References 19

I. SUPPLEMENTARY FIGURES

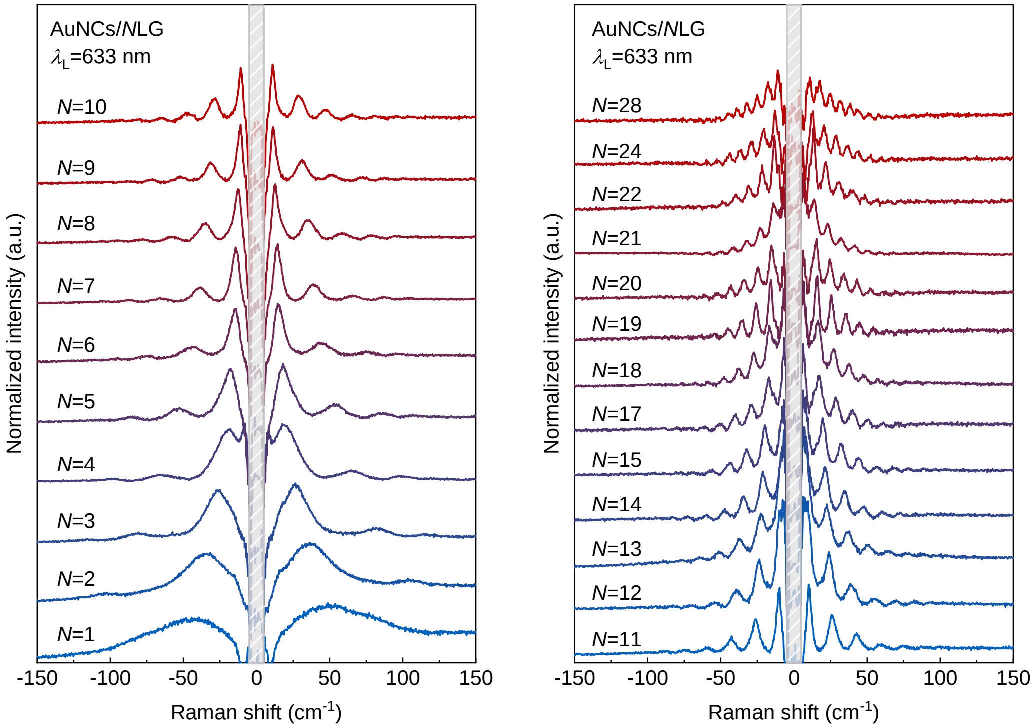

FIG. S1. Low-frequency Raman spectra of AuNCs/NLG (N=1–30) with $\lambda_{\rm L}$ = 633 nm.

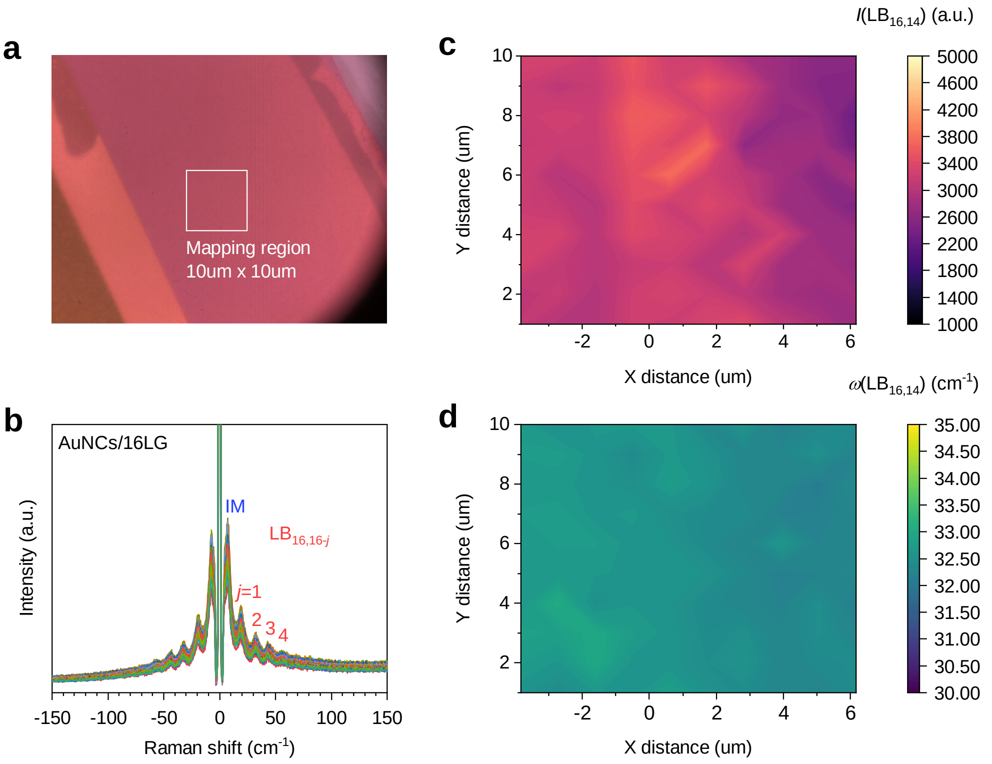

FIG. S2. (a) Optical image of the mapping region on the AuNCs/16LG sample. Mapping step size: 1 μm. (b) Low-frequency Raman spectra collected within the mapping region. (c) Raman intensity and (d) peak position mapping of $LB_{16,14}$ in AuNCs/16LG.

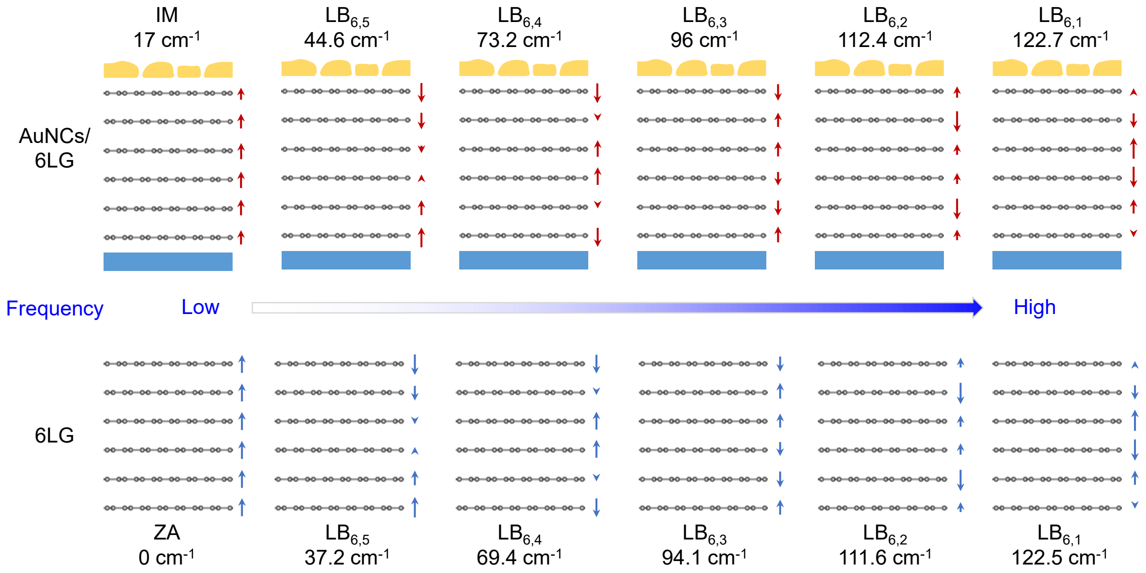

FIG. S3. Comparison of the frequencies and interlayer displacements between the out-of-plane acoustic (ZA), LB modes of 6LG and the IM, LB modes of AuNCs/6LG. The arrows indicate the displacement of each layer.

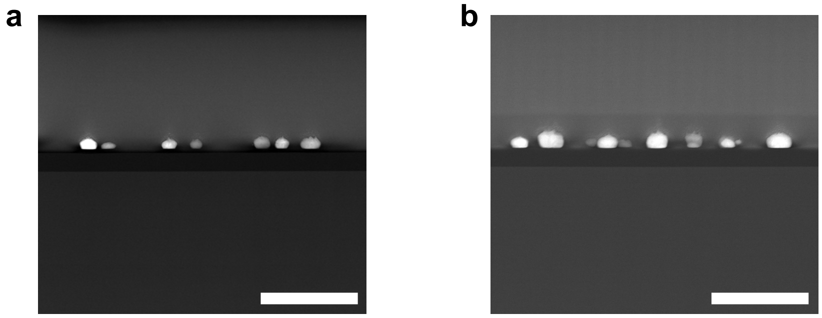

FIG. S4. TEM images of AgNCs/8LG annealed at 200°C and 400°C. Scale bars: 500 nm.

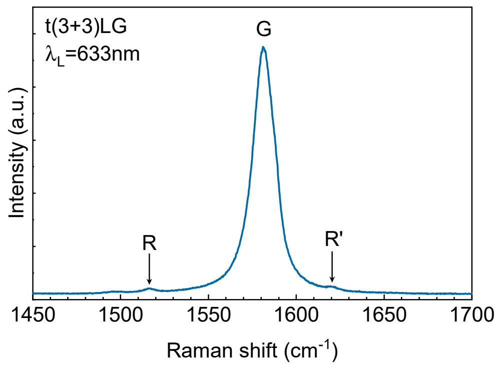

FIG. S5. High-frequency folded phonon modes of t(3+3)LG. The R (∼1516 cm^{-1}) and R′ (∼1621 cm^{-1}) modes.

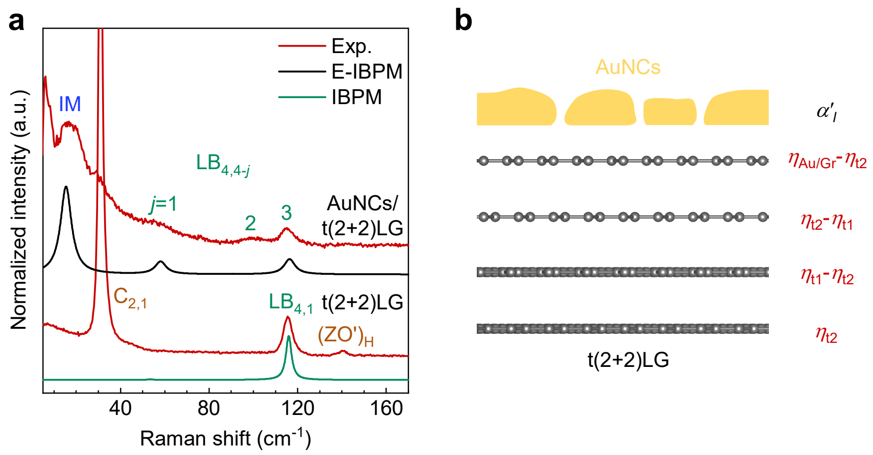

FIG. S6. (a) Experimental (Exp.) and calculated (E-IBPM or IBPM) Raman spectra of the LB modes for AuNCs/t(2+2)LG (twist angle $\theta = 10.5°$). The linewidths of the calculated LB modes in AuNCs/t(2+2)LG and t(2+2)LG are set to 6 cm^{-1} and 3 cm^{-1}, respectively. (b) Schematic diagram for interlayer bond parameters $\alpha'_{l,xx}$ of AuNCs/t(2+2)LG.

II. SUPPLEMENTARY NOTES

1. Background signal subtraction

During the Raman measurements, the signals of the low-frequency Raman modes in AuNCs/NLG are superimposed on the background signal of AuNCs. To obtain the relative intensities of the LB modes, we subtracted the background signal from the spectra of the AuNCs/NLG. Figure S7(a) compares the low-frequency Raman spectra of AuNCs and AuNCs/NLG ($N = 1, 6$ for example). The spectrum of AuNCs/NLG was normalized to its intensity maximum, while the spectra of AuNCs were scaled to overlap optimally with the featureless spectral region of AuNCs/NLG. The background-subtracted Raman spectrum of the AuNCs/NLG was then obtained by subtracting the scaled AuNCs reference spectrum from the raw AuNCs/NLG spectrum. Since the Raman signal of carriers in the SiO_2/Si substrate hindered the clear observation of interlayer modes of NLG, similar data processing procedures have been applied to the low-frequency Raman spectra of pristine NLG, as shown in Fig.S7(b).

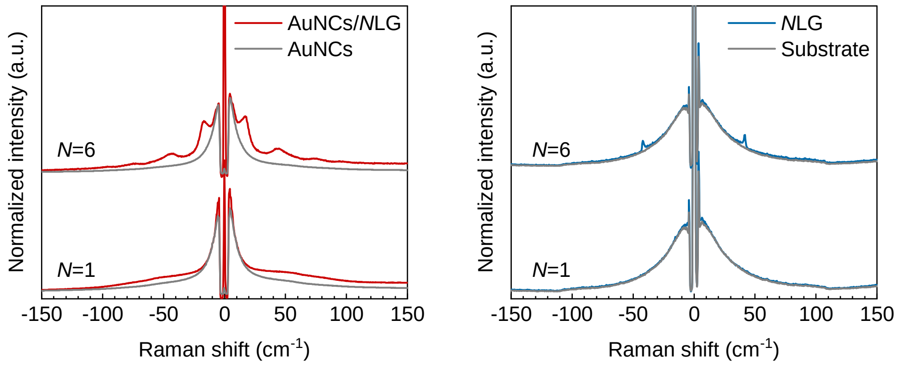

FIG. S7. Raw Raman signals and background signals of AuNCs/NLG and NLG. Low-frequency Raman spectra of (a) AuNCs/NLG and AuNCs, along with those of (b) NLG and the underlying substrate. ($N = 1, 6$).

2. Linear chain model for 2DMs coupled with AuNCs

The frequencies of LB modes in AuNCs/NLG can be calculated using the LCM. For $N > 2$, the frequencies of LB modes can be calculated by solving the following equations[1]:

$$\omega_j^2 \mathbf{M}\mathbf{u}_j = \frac{1}{4\pi c^2}\mathbf{D}\mathbf{u}_j, \tag{S1}$$

where $\mathbf{u}_j$ is the phonon eigenvector of the $\mathrm{LB}_{N,N-j}$ with frequency ω_j. $\mathbf{M}$ is the diagonal mass matrix of the NLG. $c = 3 \times 10^8$ m·s^{-1} is the speed of light. $\mathbf{D}$ is the out-of-plane interlayer force constant matrix.

Take AuNCs/4LG as an example, $\mathbf{M}$ can be written as

$$\mathbf{M} = \begin{pmatrix} m_{\mathrm{Gr}} & 0 & 0 & 0 \\ 0 & m_{\mathrm{Gr}} & 0 & 0 \\ 0 & 0 & m_{\mathrm{Gr}} & 0 \\ 0 & 0 & 0 & m_{\mathrm{Gr}} \end{pmatrix}, \tag{S2}$$

where $m_{\mathrm{Gr}} = 7.56 \times 10^{-7}$ kg·m^{-2} is the mass per unit area of graphene. $\mathbf{D}$ can be written as

$$\mathbf{D} = \begin{pmatrix} k_{\mathrm{Gr}}^{\mathrm{1st}} + k_{\mathrm{Gr}}^{\mathrm{2nd}} + k_{\mathrm{Au/Gr}} & -k_{\mathrm{Gr}}^{\mathrm{1st}} & -k_{\mathrm{Gr}}^{\mathrm{2nd}} & 0 \\ -k_{\mathrm{Gr}}^{\mathrm{1st}} & 2k_{\mathrm{Gr}}^{\mathrm{1st}} + k_{\mathrm{Gr}}^{\mathrm{2nd}} & -k_{\mathrm{Gr}}^{\mathrm{1st}} & -k_{\mathrm{Gr}}^{\mathrm{2nd}} \\ -k_{\mathrm{Gr}}^{\mathrm{2nd}} & -k_{\mathrm{Gr}}^{\mathrm{1st}} & 2k_{\mathrm{Gr}}^{\mathrm{1st}} + k_{\mathrm{Gr}}^{\mathrm{2nd}} & -k_{\mathrm{Gr}}^{\mathrm{1st}} \\ 0 & -k_{\mathrm{Gr}}^{\mathrm{2nd}} & -k_{\mathrm{Gr}}^{\mathrm{1st}} & k_{\mathrm{Gr}}^{\mathrm{1st}} + k_{\mathrm{Gr}}^{\mathrm{2nd}} + k_{\mathrm{Gr/Sub}} \end{pmatrix}. \tag{S3}$$

3. Derivation of Raman tensor elements for 2DMs coupled with AuNCs

According to the empirical bond polarizability model, the polarizability can be approximated by a sum of individual bond polarizabilities from different bonds:[2–4]

$$\alpha_{\mu\nu} = \frac{1}{2}\sum_{i,k}\left[\frac{\alpha_{k,\parallel} + 2\alpha_{k,\perp}}{3}\delta_{\mu\nu} + \left(\alpha_{k,\parallel} - \alpha_{k,\perp}\right)\left(\frac{R_{ik,\mu}R_{ik,\nu}}{|\mathbf{R}_{ik}|^2} - \frac{1}{3}\delta_{\mu\nu}\right)\right], \tag{S4}$$

where $R_{ik,\mu}$ ($R_{ik,\nu}$) is the component of the k-th bond vector $\mathbf{R}_{ik}$ connecting atom i and its neighboring atoms ($\mu, \nu = x, y, z$). $\alpha_{k,\parallel}$ and $\alpha_{k,\perp}$ are the bond polarizability for the k-th bond in the directions parallel and perpendicular to the bond, respectively.

The Raman tensor elements $\Delta\alpha_{j,\mu\nu}$ of $\mathrm{LB}_{N,N-j}$ are given by

$$\Delta\alpha_{j,\mu\nu} = \sum_{i\gamma}\left(\frac{\partial\alpha_{\mu\nu}}{\partial r_{i\gamma}}\right)_0 \Delta r_{j,i\gamma}, \tag{S5}$$

where $r_{i\gamma}$ is the position of atom i along γ direction ($\gamma = x, y, z$) in equilibrium, $(\partial\alpha_{\mu\nu}/\partial r_{i\gamma})_0$ is the derivative of the polarizability tensor element $\alpha_{\mu\nu}$ with respect to the atomic displacement from the equilibrium configuration, and $\Delta r_{j,i\gamma}$ is the eigen-displacement of atom i in $\mathrm{LB}_{N,N-j}$.

Substituting Eq.S5 into Eq.S4, the Raman tensor elements are obtained by[4]

$$\begin{aligned}\Delta\alpha_{j,\mu\nu} =& -\sum_{ik}\left\{\mathbf{R}^0_{ik}\cdot\Delta\mathbf{r}_{j,i}\left[\frac{\alpha'_{k,\parallel}+2\alpha'_{k,\perp}}{3}\delta_{\mu\nu}+\left(\alpha'_{k,\parallel}-\alpha'_{k,\perp}\right)\left(R^0_{ik,\mu}R^0_{ik,\nu}-\frac{1}{3}\delta_{\mu\nu}\right)\right]\right\}\\ &-\sum_{ik}\left\{\frac{\alpha_{k,\parallel}-\alpha_{k,\perp}}{|\mathbf{R}_{ik}|}\left[R^0_{ik,\mu}\Delta r_{j,i\nu}+R^0_{ik,\nu}\Delta r_{j,i\mu}-2R^0_{ik,\mu}R^0_{ik,\nu}\mathbf{R}^0_{ik}\cdot\Delta\mathbf{r}_{j,i}\right]\right\},\end{aligned} \tag{S6}$$

where $\mathbf{R}^0_{ik} = \mathbf{R}_{ik}/|\mathbf{R}_{ik}|$ is the equilibrium-configuration bond vector normalized to unity, $R^0_{ik,\mu}$ is the component of $\mathbf{R}^0_{ik}$. $\alpha'_{k,\parallel}$ and $\alpha'_{k,\perp}$ are the radial and normal derivatives of the bond polarizability with respect to the bond length, respectively.

For LB modes vibrating along the z direction, $\Delta\mathbf{r}_{j,i} = \Delta z_{j,i}$, which yields[4]

$$\begin{aligned}\Delta\alpha_{j,\mu\nu} =& -\sum_{ik}\left\{R^0_{ik,z}\Delta z_{j,i}\left[\frac{\alpha'_{k,\parallel}+2\alpha'_{k,\perp}}{3}\delta_{\mu\nu}+\left(\alpha'_{k,\parallel}-\alpha'_{k,\perp}\right)\left(R^0_{ik,\mu}R^0_{ik,\nu}-\frac{1}{3}\delta_{\mu\nu}\right)\right]\right\}\\ &-\sum_{ik}\left\{\frac{\alpha_{k,\parallel}-\alpha_{k,\perp}}{|\mathbf{R}_{ik}|}\left[R^0_{ik,\mu}\Delta z_{j,i}\delta_{\nu z}+R^0_{ik,\nu}\Delta z_{j,i}\delta_{\mu z}-2R^0_{ik,\mu}R^0_{ik,\nu}R^0_{ik,z}\Delta z_{j,i}\right]\right\}\end{aligned} \tag{S7}$$

Therefore, $\Delta\alpha_{j,xx}$ is given by

$$\begin{aligned}\Delta\alpha_{j,xx} =& -\sum_{ik}\left\{\left[\alpha'_{k,\perp}+\left(\alpha'_{k,\parallel}-\alpha'_{k,\perp}\right)\left(R^0_{ik,x}\right)^2-2\frac{\alpha_{k,\parallel}-\alpha_{k,\perp}}{|\mathbf{R}_{ik}|}\left(R^0_{ik,x}\right)^2\right]R^0_{ik,z}\Delta z_{j,i}\right\}\\ =&\sum_{ik}\eta_{ik,x}R^0_{ik,z}\Delta z_{j,i},\end{aligned} \tag{S8}$$

and $\Delta\alpha_{j,zz}$ is given by

$$\begin{aligned}\Delta\alpha_{j,zz} =& -\sum_{ik}\left\{\left[\alpha'_{k,\perp}+\left(\alpha'_{k,\parallel}-\alpha'_{k,\perp}\right)\left(R^0_{ik,z}\right)^2-2\frac{\alpha_{k,\parallel}-\alpha_{k,\perp}}{|\mathbf{R}_{ik}|}\left[1-\left(R^0_{ik,z}\right)^2\right]\right]R^0_{ik,z}\Delta z_{j,i}\right\}\\ =&\sum_{ik}\eta_{ik,z}R^0_{ik,z}\Delta z_{j,i}.\end{aligned} \tag{S9}$$

Here, $\eta_{ik,x}$ and $\eta_{ik,z}$ are parameters related to the x and z components of bond vector $\mathbf{R}_{ik}$, respectively. For 2DMs and vdWHs, the intralayer bonds are not compressed or stretched during interlayer vibrations, and thus do not contribute to the change of the polarizability. Only the interlayer bonds are altered during such a vibration, leading to the polarizability

change. Since the atoms in the same layer have the same displacements, the bonds between atoms in adjacent layers can be simplified as one 'interlayer bond', and for the l-th layer only two interlayer bonds (η_l and η_{l+1}) need to be considered. Therefore, the Eqs.S8 and S9 can be written as

$$\Delta\alpha_{j,xx} = \sum_l (\eta_{l,x} - \eta_{l+1,x})\Delta z_{j,l}, \tag{S10}$$

and

$$\Delta\alpha_{j,zz} = \sum_l (\eta_{l,z} - \eta_{l+1,z})\Delta z_{j,l}. \tag{S11}$$

4. Possible enhancement effect of LB modes in AuNCs/NLG by out-of-plane components of local fields

In specific PERS scenarios, the enhancement of out-of-plane modes is predominantly attributed to the out-of-plane field component of plasmonic near-fields. This has been observed in systems such as MoS_2 coupled with nanoparticle-on-mirror (NPOM) nanoantennas and the silicene in tip-enhanced Raman scattering (TERS) setup[5, 6]. The out-of-plane component of local electric field (E^z_{Loc}) generated by these metallic nanostructures is significantly stronger than the in-plane component ($E_{\mathrm{Loc},x}$) by several orders of magnitude. Furthermore, the experimental setups in these studies facilitate the detection of Raman signals induced by $E_{\mathrm{Loc},z}$. For instance, the A^1 mode of silicene is enhanced by $E_{\mathrm{Loc},z}$, following the relation $I(A^1) \propto |\Delta\alpha_{zz} g^2_{zz}|$[5]. Here, $\Delta\alpha_{zz}$ is the zz component of Raman tensor of A^1 mode, and g_{zz} is the zz component of the electric field enhancement tensor[5].

In our work, the high numerical aperture (0.9 NA) objective also allows for the detection of PERS signals of LB modes enhanced by $E_{\mathrm{Loc},z}$. In this case, the $\mathrm{LB}_{N,N-j}$ intensity is related to the zz component of the Raman tensor ($\Delta\alpha_{j,zz}$) and the z component of the local electric field at each graphene layer ($E_{\mathrm{Loc},l,z}$). According to Eq.S9, $\Delta\alpha_{j,zz} = \sum_l \alpha'_{l,zz}\Delta z_{j,l} = \sum_l (\eta_{l,z} - \eta_{l+1,z})\Delta z_{j,l}$, and thus the intensity of $\mathrm{LB}_{N,N-j}$ can be expressed as

$$I_{j,zz} \propto \frac{n_j + 1}{\omega_j} \left| \sum_l (\eta_{l,z} - \eta_{l+1,z})\Delta z_{j,l} E^2_{\mathrm{Loc},l,z} \right|^2. \tag{S12}$$

For AuNCs/NLG, the interlayer bonds at AuNCs/graphene interface and interior interlayer bonds in NLG are different, whose corresponding bond parameters are considered as $\eta_{\mathrm{Au/Gr},z}$ and $\eta_{\mathrm{Gr},z}$, respectively. Similar to the case in the main text, only the 1st and the N-th

graphene layers have non-zero $\alpha'_{l,zz}$, i.e., $\alpha'_{1,zz} = \eta_{\mathrm{Au/Gr},z} - \eta_{\mathrm{Gr},z}$ and $\alpha'_{N,zz} = \eta_{\mathrm{Gr},z}$. Therefore, Eq.S12 can be simplified as

$$I_{j,zz} \propto \frac{n_j + 1}{\omega_j} \left| \left(\eta_{\mathrm{Au/Gr},z} - \eta_{\mathrm{Gr},z}\right) \Delta z_{j,l} \cdot E^2_{\mathrm{Loc},1,z} + \eta_{\mathrm{Gr},z} \Delta z_{j,1} \cdot E^2_{\mathrm{Loc},N,z} \right|^2 . \tag{S13}$$

FDTD simulations reveal that the plasmon-induced local electric fields generated by AuNCs exhibit comparable magnitudes for all three vector components ($E_{\mathrm{Loc},1,x}$, $E_{\mathrm{Loc},1,y}$, $E_{\mathrm{Loc},1,z}$) in the first graphene layer, as depicted in Fig.S8. For a large N, $E_{\mathrm{Loc},N,z}/E_{\mathrm{Loc},1,z} \approx 0$, which leads to a further simplification of Eq.S13 as

$$I_{j,zz} \propto \frac{n_j + 1}{\omega_j} \left| \Delta z_{j,1} \right|^2 . \tag{S14}$$

Eq.S14 suggests that the intensity profile of LB modes contributed from $E_{\mathrm{Loc},z}$ is similar to that contributed from $E_{\mathrm{Loc},x}$.

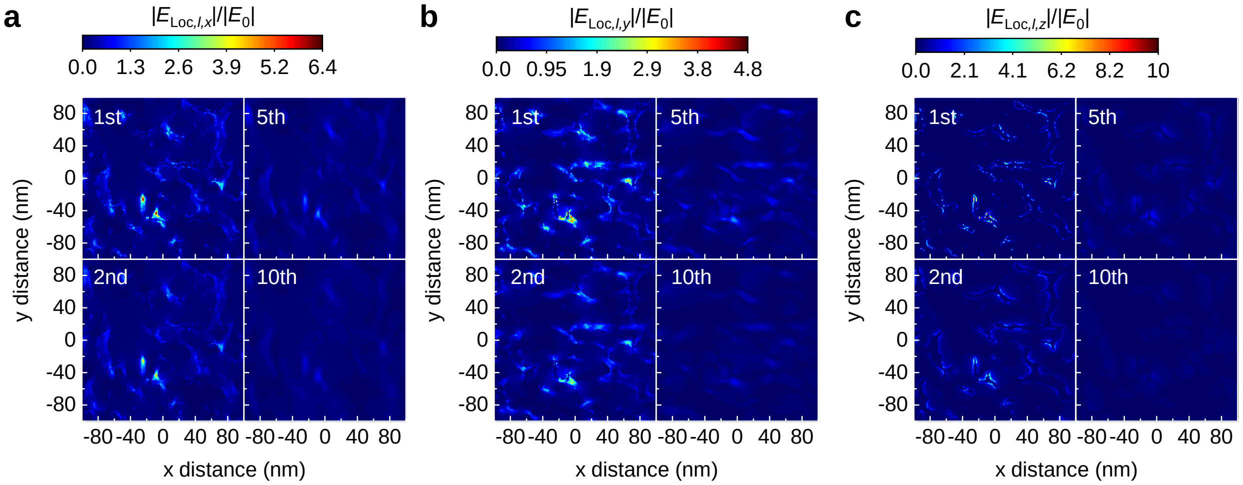

FIG. S8. Simulated distribution of (a) x-, (b) $y-$, and (c) $z-$components of local electric field at 1st, 2nd, 5th and 10th graphene layers adjacent to AuNCs.

5. Plasmon-enhanced LB modes in NLG coupled with AuNCs with different shapes and densities

The PERS of LB modes in AuNCs/2DM systems is quantitatively described by the E-IBPM (Equation 5 in the main text). This is a new theoretical framework incorporating both local field enhancement along the out-of-plane direction and polarizability modulation,

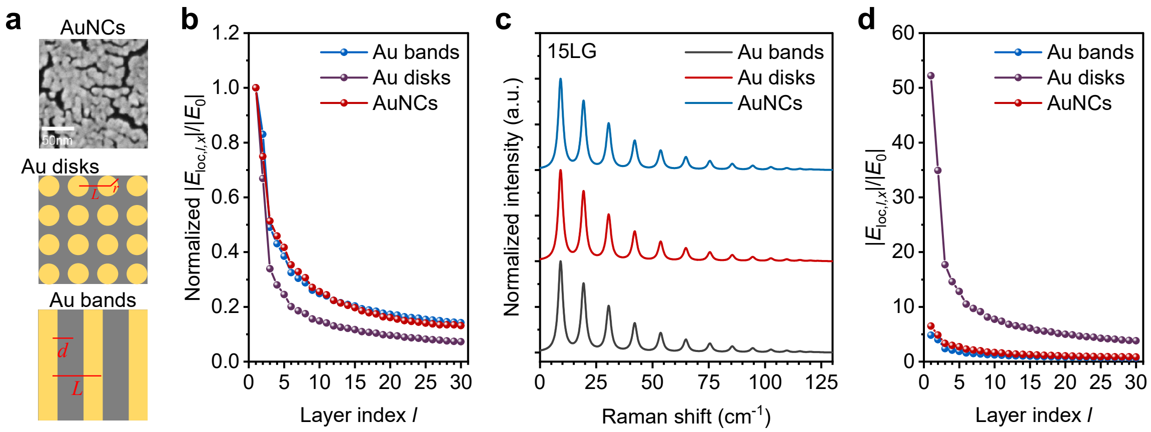

FIG. S9. (a) SEM image of the prepared Au nanofilm, schematic diagrams of Au disk (L=140 nm, r=50 nm, thickness=20 nm) and Au bands (d=50 nm, L=75 nm, thickness=20 nm). With these set variables, the plasmonic particle content for the above three cases is the same. (d) Simulated distribution of normalized x-component of the maximum local field ($|E_{\mathrm{Loc},l,x}|/|E_0|$) in different graphene layers near Au plasmonic particles with different shapes by the FDTD method. (e) Simulated Raman spectra by considering different maximum $|E_{\mathrm{Loc},l,x}|/|E_0|$ for three types of plasmonic particles. (f) Simulated distribution of maximum $|E_{\mathrm{Loc},l,x}|/|E_0|$ for the above three kinds of plasmonic particles.

in which the former is related to the local plasmonic field in homogeneously extending into the 2DMs while the latter is associated with the interfacial interaction between 2DMs and plasmonic NCs. Since the interfacial interaction is dominated by the material-specific properties of the 2DMs and the plasmonic metal, the resulting polarizability modulation is largely independent of the nanoparticle shape. Consequently, variations in polarizability modulation arising from differences in nanoparticle shape can be considered negligible. In this case, in the Equation 5 of the main text, the k-th interlayer bond parameter related to the x component of the interlayer bond polarizability and the displacement of the l-th layer for one specific LB mode are the same for AuNCs with different shapes and distributions. Thus, the relative intensity of the LB modes is dictated by the relative magnitude of the plasmonic field at each layer (*i.e.*, the decay of the plasmonic field across layers). In contrast, the absolute Raman intensity scales with the field's absolute strength.

Figure S9(a,b) shows the simulated distribution of the normalized x-component of the maximum local field ($|E_{\mathrm{Loc},l,x}|/|E_0|$) in different graphene layers near plasmonic particles cor-

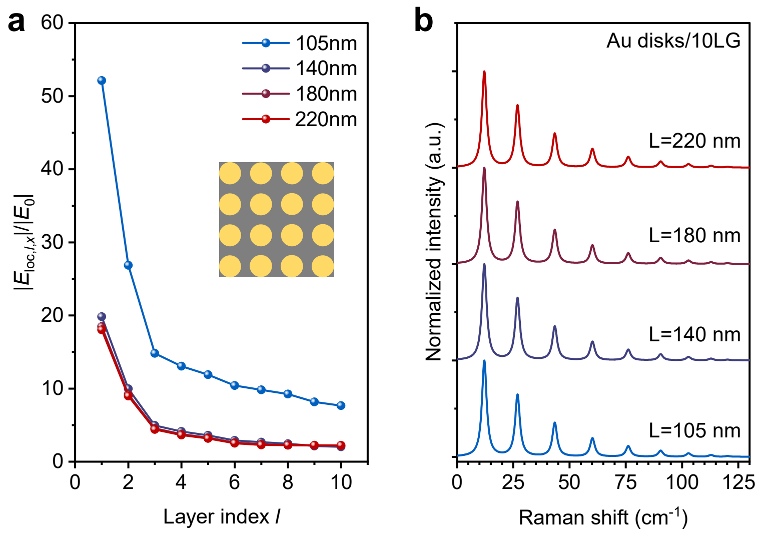

FIG. S10. (a) Simulated maximum $|E_{\text{Loc},l,x}|/|E_0|$ in different graphene layers near Au nanodisks with different densities by considering different distances between the adjacent disks (L=105, 140, 180, 220 nm, r=50 nm, thickness=8 nm) by the FDTD method. (b) Simulated Raman spectra by considering different maximum $|E_{\text{Loc},l,x}|$ for three types of AuNCs.

responding to AuNCs from the SEM image, Au disk and Au bands by the FDTD method (considering the same AuNCs content for three cases). A decay of maximum $|E_{\text{Loc},l,x}|/|E_0|$ with increasing layer index l can be observed in three cases. In addition, the normalized maximum $|E_{\text{Loc},l,x}|/|E_0|$ shows similar decay in AuNCs from SEM image and Au bands, while that of Au disks exhibits a much more rapid decay. According to the Equation 5 in the main text, this should result in similar relative Raman intensity profile of the LB modes for 2DMs coupled to plasmonic particles with different shapes. Indeed, when we put the simulated maximum $|E_{\text{Loc},l,x}|$ for different shaped plasmonic particles into the Equation 5, the calculated Raman spectra (Fig.S9(c)) for 15LG coupled with AuNCs, Au disks, and Au bands are quite similar. In contrast, the intensity profiles of the maximum $|E_{\text{Loc},l,x}|/|E_0|$ (Fig.S9(d)) show significant differences for different shaped plasmonic particles, which suggests that the enhanced LB mode intensity greatly varies with AuNCs shapes. Remarkably, the maximum $|E_{\text{Loc},l,x}|/|E_0|$ values of the AuNCs fabricated by thermal evaporation proposed in this work is significant, close to that of Au bands but smaller than that of Au disks. This suggests that the AuNCs in this work should show intense PERS intensity for the LB modes, as confirmed by the Raman spectra with good signal-to-noise ratio in the

manuscript. Therefore, the proposed plasmonic cavities of AuNCs used in the manuscript provide a strong and well-characterized plasmonic field ideal for demonstrating the core principle of PERS for LB modes.

In principle, different NCs densities result in different plasmonic field absolute magnitude, but not a significant change in the field's decay across the layers of the 2DMs. For a controlled discussion, we take Au disks as an example. Indeed, by varying the distance between the nanodisk (with radius of 50 nm) from 105 to 260 nm, the simulated maximum $|E_{\mathrm{Loc},l,x}|/|E_0|$ show similar field decay but distinguished field magnitude, as shown in Fig.S10(a). The corresponding simulated Raman spectra (Fig.S10(b)) show negligible difference for different densities of AuNCs. This suggests that for different NC densities, the plasmon-enhanced LB modes exhibit similar relative Raman intensity profiles, while greater enhanced Raman intensities for LB modes with larger NC densities as the number of hotspots in the laser-illuminated region increases with NC densities. Notably, at low NC densities, the overall Raman signal from the sample would be lower because a smaller fraction of the laser spot is covered by enhancing hotspots. This would result in a lower signal-to-noise ratio, making the LB modes harder to detect experimentally. However, at the locations where enhancement does occur, the E-IBPM would still accurately describe the relative intensity and behavior of the enhanced LB modes. The model explains the quality of the enhancement at a point, while the density affects the quantity of the signal collected over an area.

To thoroughly address this concern and demonstrate the broad applicability of our proposed system, we have conducted additional experiments by preparing NLG coupled with different plasmonic cavities, i.e., as-prepared AuNCs, AuNCs annealed at 200°C, 600°C, as-prepared AgNCs, AgNCs annealed at 200°C and 400°C, which show different shapes and distributions of plasmonic cavities as determined by the SEM images (Fig.??(a)). The corresponding dark-field scattering spectra (Fig.??(b)) are quite different for these plasmonic cavities, where the AuNCs show a significant resonance peak at around 650 nm, while the AgNCs show a broad resonance band ranging from 500–700 nm. The Raman spectra under the resonance of the plasmonic cavities were measured using a 633 nm laser for both AuNCs and AgNCs, as illustrated in Fig.??(c). The plasmon-enhanced LB modes of NLG are significant in all these different systems, whose peak positions show differences due to the different interfacial coupling force constants between Au and NLG, for NLG coupled with AuNCs ($k_{\mathrm{Au/Gr}}$=0.3k_{Gr}) and AgNCs ($k_{\mathrm{Ag/Gr}}$=0.1k_{Gr}), in which $k_{\mathrm{Au/Gr}}$, $k_{\mathrm{Ag/Gr}}$ and k_{Gr}

are the interfacial force constants for Au/NLG and Ag/NLG interfaces and interlayer force constant between adjacent graphene layers, respectively. The frequencies of these plasmon-enhanced LB modes can be well reproduced by the LCM. By applying the E-IBPM with the frequency and layer displacements of each plasmon-enhanced LB mode determined by the LCM, the relative Raman intensity of these LB modes from experiments can be well understood, as elucidated in Fig.??(c). This further validates the reproducibility and applicability of proposed method and E-IBPM for NLG coupled with various plasmonic cavities in different shapes and distributions.

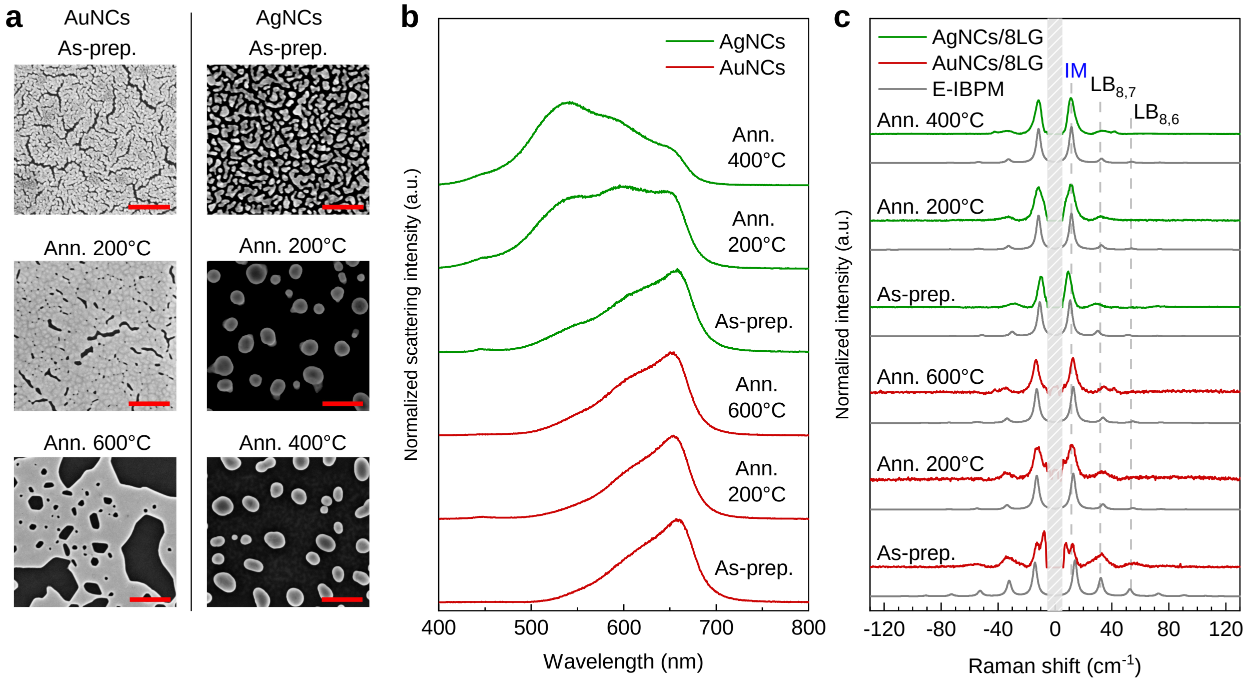

FIG. S11. (a) SEM images of as-prepared AuNCs, AuNCs annealed at 200°C, 600°C, as-prepared AgNCs, AgNCs annealed at 200°C and 400°C, (b) the corresponding dark-field scattering spectra, (c) the corresponding experimental Raman spectra along with the simulated ones by E-IBPM.

6. Determine the layer number of hBN and LB force constants in AuNCs/NL-hBN structure

For the hBN samples, a combination of AFM and Raman spectra of plasmon-enhanced LB modes of AuNCs/hBN provides a reliable method for determining thickness. The inset of Fig.S12(a) shows the atomic force microscopy (AFM) topography of an hBN sample

before thermally evaporation of AuNCs. A step-height analysis confirmed its thickness of approximately 33 layers (11.2 nm). And the top spectrum in Fig.S12(a) shows the corresponding low-frequency Raman spectrum measured from this sample after coupling with the AuNCs. Critically, this layer number (N=33) was further confirmed by our LCM analysis. The calculated LBM frequencies for N=33 using the LCM are in excellent agreement with the experimental peak positions observed in Fig.S12(b).

The layer displacements involved in the simulation of LB modes in AuNCs/NL-hBN can be calculated by LCM provided that the LB force constants are known. These force constants can be fitted by analyzing the N-dependence of LB modes in AuNCs/NL-hBN. Figure S12(a) shows the low-frequency Raman spectra of AuNCs/NL-hBN with $\lambda_{\rm L}$ = 633 nm and N ranging from 16 to 33. The N of NL-hBN was determined by AFM, and the extracted frequencies of LB modes are summarized in Fig.S12(b). In the LCM of AuNCs/NL-hBN, the LB force constant of hBN is known to be $k_{\rm hBN} = 9.9 \times 10^{19}$ N$\cdot$m^{-3}[1], and the ones at the AuNCs/hBN interface ($k_{\rm Au/hBN}$) and at hBN/substrate interface ($k_{\rm hBN/Sub}$) are fitting parameters, which are finally determined as $k_{\rm Au/hBN} = 0.1k_{\rm hBN}$ and $k_{\rm hBN/Sub} = 2k_{\rm hBN}$. The calculated frequencies of LB modes in AuNCs/NL-hBN based on LCM with these LB force constants are also shown in Fig.S12(b), which matches well with the experimental results. This good agreement further confirms the determination of hBN thickness and the LB force constants in AuNCs/NL-hBN structure.

7. Simulation of LB modes in t$(m+m)$LG based on IBPM

In conventional Raman scattering, the Raman intensity of the j-th phonon mode in 2DMs and vdWHs can be calculated by

$$I_j \propto \frac{n_j+1}{\omega_j} \left|\mathbf{e}_{\rm s}^{\dagger} \cdot \Delta\alpha_j \cdot \mathbf{e}_{\rm i}\right|^2, \tag{S15}$$

where ω_j is the mode frequency, $\mathbf{e}_{\rm i}$ and $\mathbf{e}_{\rm s}$ are the polarization of incident and scattered light. In $z(xx)\bar{z}$ scattering geometry,

$$I_j \propto \frac{n_j+1}{\omega_j} \left|\Delta\alpha_{j,xx}\right|^2. \tag{S16}$$

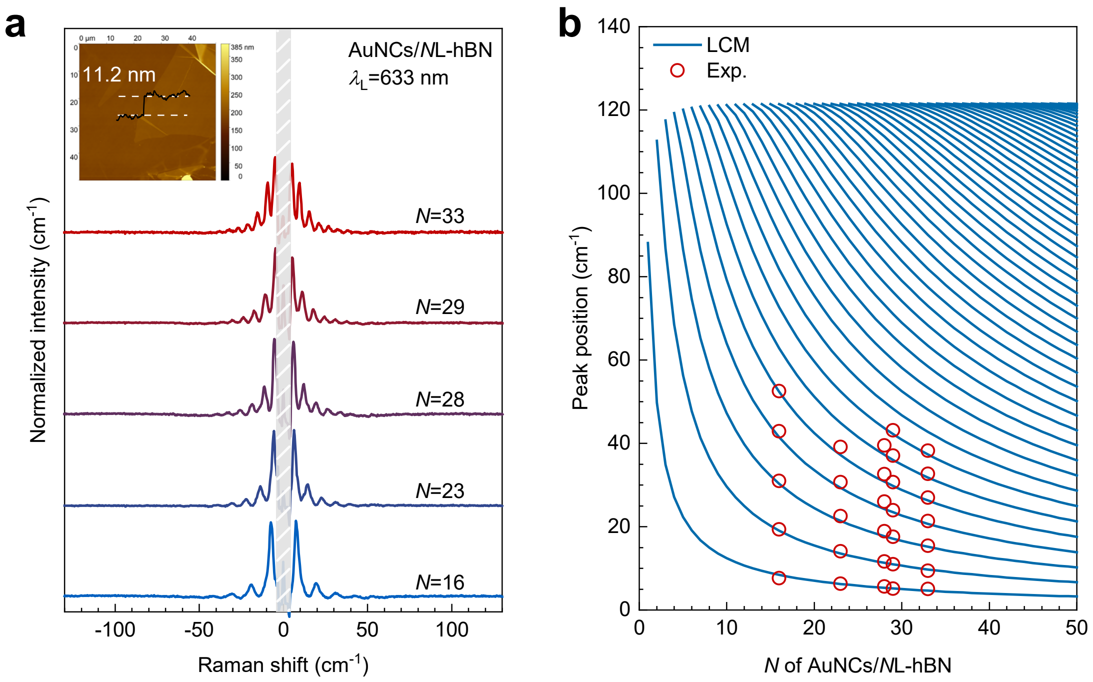

FIG. S12. (a) Low-frequency Raman spectra of 16L-hBN and AuNCs/NL-hBN. The inset shows the AFM topography of an hBN sample with thickness of 11.2 nm. (b) Extracted frequencies of LB modes as a function of N from (a), along with the fitting results using the LCM.

$\Delta\alpha_{j,xx}$ can be obtained from Eq.S8. Therefore, the intensity of $\mathrm{LB}_{N,N-j}$ can be calculated by

$$I_j \propto \frac{n_j+1}{\omega_j}\left|\sum_l(\eta_{l,x}-\eta_{l+1,x})\Delta z_{j,l}\right|^2. \tag{S17}$$

$\Delta z_{j,l}$ can be calculated by LCM with the same LB force constants used in NLG. Since the t(m+m)LG samples were fabricated on substrates without plasma treatment, the interaction between sample and substrate can be neglected.

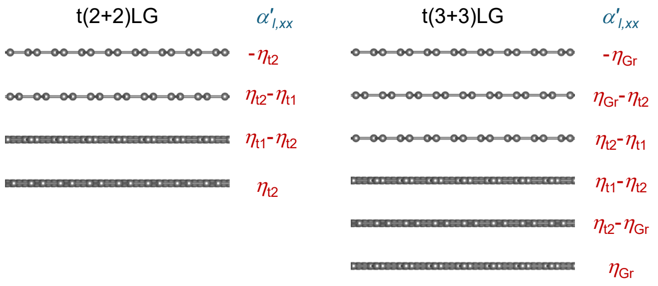

FIG. S13. Schematic of t$(m+m)$LG ($m=2,3$) with twist angle of 10.5° and $\alpha'_{l,xx}$ at each layer.

The $\alpha'_{i,xx} = \eta_{l,x} - \eta_{l+1,x}$ for the l-th layer in t(2+2)LG and t(3+3)LG are illustrated in Fig.S13. In these cases, $\eta_{\rm t1} = -1$, $\eta_{\rm t2} = 0.8$ and $\eta_{\rm Gr} = 0.01$. The experimental Raman spectrum of t(2+2)LG and t(3+3)LG with $\lambda_{\rm L} = 633$ nm and the corresponding simulated LB modes using Eq.S17 are shown in Fig.S6(a) and Fig.4(b), respectively.

[1] Wu, H. et al. Probing the interfacial coupling in ternary van der Waals heterostructures. *npj 2D Materials and Applications* **6**, 87 (2022).

[2] Saito, R. et al. Raman spectra of graphene ribbons. *Journal of Physics: Condensed Matter* **22**, 334203 (2010).

[3] Luo, X. et al. Stacking sequence determines Raman intensities of observed interlayer shear modes in 2D layered materials – A general bond polarizability model. *Journal of Physics: Condensed Matter* **5**, 14565 (2015).

[4] Liang, L. B. et al. Interlayer bond polarizability model for stacking-dependent low-frequency Raman scattering in layered materials. *Nanoscale* **9**, 15340-15355 (2017).

[5] Sheng, S. et al. Vibrational properties of a monolayer silicene sheet studied by tip-enhanced Raman spectroscopy. *Physical Review Letters* **119**, 196803 (2017).

[6] Chen, W. et al. Probing the limits of plasmonic enhancement using a two-dimensional atomic crystal probe. *Light: Science & Applications* **7**, 56 (2018).
